# Integrative profiling of lung cancer biomarkers EGFR, ALK, KRAS, and PD-1 with emphasis on nanomaterials-assisted immunomodulation and targeted therapy

**DOI:** 10.3389/fimmu.2025.1649445

**Published:** 2025-08-25

**Authors:** Li Peng, Hongmei Li, Wencai Yan, Yingjie Liu, Haihao Zhu, Yingcai Hong

**Affiliations:** ^1^ Chongqing Emergency Medical Center, Chongqing, China; ^2^ Shanghai Labway Clinical Laboratory, Shanghai, China; ^3^ Jiangsu Danyang Traditional Chinese Medicine Hospital, Danyang, Jiangsu, China; ^4^ Department of Thoracic Surgery, Shenzhen People's Hospital (The First Affiliated Hospital, Southern University of Science and Technology; The Second Clinical Medical College, Jinan University), Shenzhen, Guangdong, China

**Keywords:** lung cancer biomarkers, structural bioinformatics, molecular docking, transcriptomic validation, precision oncology

## Abstract

**Background:**

Lung cancer remains the leading cause of cancer-related mortality globally, primarily due to late-stage diagnosis, molecular heterogeneity, and therapy resistance. Key biomarkers such as EGFR, ALK, KRAS, and PD-1 have revolutionized precision oncology; however, comprehensive structural and clinical validation of these targets is crucial to enhance therapeutic efficacy.

**Methods:**

Protein sequences for EGFR, ALK, KRAS, and PD-1 were retrieved from UniProt and modeled using SWISS-MODEL to generate high-confidence 3D structures. Protein–protein interaction (PPI) networks were constructed via STRING to explore functional associations and signaling networks. Molecular docking using SwissDock evaluated the binding affinities of established inhibitors. Transcriptomic validation was conducted using RNA-seq datasets from GEPIA2, TNMplot, and UALCAN to assess differential expression and clinical subgroup relevance. Experimental validation was performed via qRT-PCR in NSCLC cell lines (A549, H1975, H520).

**Results:**

Robust 3D models were obtained, with MolProbity scores between 0.67 and 2.09, confirming structural reliability. Key mutations, including EGFR T790M and KRAS G12C, were localized to ATP-binding clefts and allosteric pockets respectively, based on structural mapping using SWISS-MODEL. PPI analysis revealed EGFR’s integration into ERBB and MAPK/PI3K pathways, ALK’s fusion-driven activation via EML4 and PI3K-AKT signaling, KRAS’s links to MAPK effectors, and PD-1’s interaction with immune checkpoint ligands PD-L1/PD-L2. Docking results showed strong EGFR–Gefitinib affinity (−5.94 kcal/mol, Kd 4.38 × 10^-5^ M), while KRAS inhibitors Adagrasib and Sotorasib demonstrated moderate binding (−3.94 and −3.72 kcal/mol, respectively). Transcriptomic analyses revealed significant overexpression of EGFR (2.8-fold), KRAS (2.3-fold), ALK (1.9-fold), and PDCD1 (2.1-fold) in NSCLC tissues (p < 0.01). qRT-PCR corroborated these findings, with H1975 cells displaying elevated EGFR (3.2-fold) and KRAS (2.4-fold), and H520 cells showing increased ALK (2.7-fold) and moderate PDCD1 expression.

**Conclusion:**

This integrative study combining structural modeling, molecular interaction analysis, and transcriptomic validation confirms EGFR, ALK, KRAS, and PD-1 supports their relevance as clinically actionable and structurally druggable biomarkers in NSCLC. These findings support their continued use in targeted therapy design and precision diagnostics, highlighting nanomaterials as ideal carriers due to their ability to enhance immune checkpoint blockade and drug bioavailability in NSCLC.

## 1 Introduction

Lung cancer remains the leading cause of cancer-related mortality worldwide, accounting for an estimated 2.21 million new cases and 1.8 million deaths in 2020, which represents 11.4% of all new cancer diagnoses and 18% of all cancer deaths, according to GLOBOCAN data (1). This burden is projected to rise to over 3.6 million new cases annually by 2040, largely driven by population ageing, continued tobacco use in developing regions, rising urban pollution, and sustained exposure to occupational and environmental carcinogens (2, 3). While cigarette smoking remains the predominant risk factor—linked to approximately 87% of global lung cancer cases—the incidence among never-smokers is rising significantly, especially in East Asia, where up to 60% of lung cancer cases in women occur in non-smokers (4–6). Additional contributors include radon exposure, ambient particulate matter (PM2.5), second-hand smoke, occupational hazards such as asbestos and silica, and genetic predispositions through polymorphisms or germline mutations (7). Despite advances in treatment, the overall five-year survival rate remains dismal at around 19.8%, dropping to below 5% for stage IV disease, compared to over 60% for early-stage, localized tumors (8). More than 70% of patients are diagnosed at an advanced stage, reflecting the inadequacy of symptom-based diagnosis and limitations of current screening such as low-dose CT, thus underscoring the need for molecularly precise diagnostics (9). This underscores the urgent need for early, non-invasive, and molecularly precise diagnostic approaches. Molecular biomarkers have transformed the landscape of lung cancer treatment, enabling targeted and personalized therapeutic strategies. Key biomarkers such as *EGFR*, *ALK*, *KRAS*, and *PD-1* have been instrumental in guiding tyrosine kinase inhibitors and immune checkpoint blockade therapies (10, 11). *EGFR* mutations, common in non-smokers and East Asian populations, predict response to TKIs like Osimertinib (12). *ALK* rearrangements, prevalent in younger, non-smoking patients, are responsive to agents like crizotinib and alectinib. *KRAS* mutations, particularly G12C, represent historically “undruggable” targets that are now actionable with inhibitors such as sotorasib. Immunotherapy, particularly targeting *PD-1/PD-L1*, has revolutionized lung cancer management, offering durable responses in biomarker-selected populations (13). Nanomaterials have emerged as transformative tools in immunotherapy due to their ability to enhance pharmacokinetics, target tumor microenvironments, and modulate immune checkpoint responses at nanoscale resolution. Recent advancements in cancer nanomedicine have enabled the targeted delivery of immunomodulatory agents using functionalized nanomaterials. Nanoparticles engineered to interfere with the PD-1/PD-L1 axis offer the potential to overcome immune evasion mechanisms in NSCLC (14). Understanding the structural architecture of PD-1 and associated immune ligands is crucial for designing nanoscale immune checkpoint inhibitors and for developing hybrid diagnostic-therapeutic platforms. However, the structural heterogeneity and dynamic mutation landscape of these biomarkers necessitate deeper investigation into their conformational properties, interaction networks, and clinical expression patterns. This study adopts a structure-guided and expression-validated approach to characterize *EGFR*, *ALK*, *KRAS*, and *PD-1*—four of the most clinically actionable targets in lung cancer. Using homology modeling via SWISS-MODEL, we constructed high-fidelity three-dimensional representations to identify key functional domains, mutation hotspots, and drug-binding interfaces. Complementing this, molecular docking was employed to evaluate inhibitor interactions, and protein–protein interaction (PPI) analysis via STRING provided insights into pathway integration and oncogenic networks. Importantly, we validated the clinical relevance of these targets using transcriptomic expression data from public qPCR datasets (GEPIA2, UALCAN, TNMplot), confirming their differential expression in tumor versus normal tissues. By integrating structural bioinformatics with molecular and expression data, this study aims to reinforce the role of *EGFR*, *ALK*, *KRAS*, and *PD-1* as precision targets in lung cancer, providing a foundation for future diagnostics, therapeutic design, and personalized oncology strategies (15).

## 2 Methods and materials

This study was conducted in compliance with the Declaration of Helsinki and was approved by the Institutional Ethics Committee of Shenzhen People’s Hospital, Shenzhen communicate through letter no. LL-KY-2024162-01. All analyses were performed using publicly available bioinformatics databases web-based tools and human-derived cells but without the use of human or animal experimental subjects.

### 2.1 Accession of protein sequences

The amino acid sequences of four lung cancer biomarkers—EGFR (P00533), ALK (Q9UM73), KRAS (P01116), and PD-1 (PDCD1, Q15116)—were retrieved in FASTA format from the UniProt Knowledgebase (https://www.uniprot.org/) between December 2024 and May 2025. These targets were selected based on their high clinical relevance in non-small cell lung cancer (NSCLC) and their roles in therapy resistance, tumor progression, or immune evasion. Additional biomarkers reviewed during selection are listed in **Supplementary Table S1**.

### 2.2 Homology modeling via SWISS-MODEL

SWISS-MODEL (server- https://swissmodel.expasy.org/, version updated January 2024) was selected for its template-based precision and user-defined control, which was essential for mutation-specific modeling; AlphaFold’s models, while highly accurate, do not currently allow mutation-guided customizations or ligand-ready conformations. Template identification was carried out using BLAST and HHblits searches against the SWISS-MODEL Template Library (SMTL). The best-fit templates were selected based on sequence identity, template resolution, and structural coverage. Each model was evaluated by Global Model Quality Estimation (GMQE) and QMEAN Z-score. Final models were downloaded in PDB format between January and April 2025 and were used for mutation mapping and binding-site prediction (16, 17).

### 2.3 Molecular docking

Protein-ligand docking was conducted using SwissDock (http://www.swissdock.ch/, accessed April 2025), a web server based on the EADock DSS docking engine and the CHARMM22 force field. Ligands for docking included clinically approved inhibitors: gefitinib (EGFR), crizotinib and alectinib (ALK), sotorasib and adagrasib (KRAS), and a PD-1 small molecule analogue. Blind docking was employed to explore allosteric and orthosteric binding across the protein surface. Docked conformations were scored based on FullFitness and ΔG (binding free energy) values. Interaction analyses were performed using PyMOL v2.5 and BIOVIA Discovery Studio Visualizer v21.1, with hydrogen bond distances and van der Waals overlaps cross-validated against known crystal structures (RMSD ≤ 2 Å where applicable) (18).

### 2.4 Protein-protein interaction network construction

To evaluate the functional interaction landscape of each biomarker, protein–protein interaction (PPI) networks were constructed using the STRING database (https://string-db.org/, version 12.0). Each protein (EGFR, ALK, KRAS, PDCD1) was individually queried, and results were filtered with a high-confidence interaction score cutoff of ≥0.90. Interactions were derived from curated databases, experimental evidence, co-expression, text mining, and co-occurrence. PPI networks were accessed and downloaded in February 2025. The top 10 functional partners for each protein were selected for interpretation and integration into biological pathways (19).

### 2.5 Transcriptomic expression validation

To validate the relevance of the selected biomarkers at the transcriptomic level, a qPCR-style in silico gene expression analysis was performed using the following public RNA-seq data platforms: GEPIA2 (http://gepia2.cancer-pku.cn/; accessed March 2025): A TCGA–GTEx portal used for tumor vs. normal differential expression. Statistical cutoffs were set at |log_2_FC| ≥ 1 and *p* < 0.01. Data were analyzed for LUAD and LUSC cohorts. TNMplot (https://www.tnmplot.com/; accessed April 2025): Used for cross-platform (RNA-seq and microarray) validation of EGFR, ALK, KRAS, and PDCD1 expression in NSCLC versus normal tissues. Consistency across datasets strengthened reliability. UALCAN (http://ualcan.path.uab.edu/; accessed April 2025): Provided stage-wise and subtype-specific analysis of biomarker mRNA expression. Promoter methylation data indicated transcriptional activation rather than repression for EGFR, ALK, and KRAS, suggesting their overexpression is driven by oncogenic signaling rather than epigenetic deregulation **Supplementary Table S2**, mimicking a qPCR-style output with log_2_ fold changes, p-values, and clinical relevance. For laboratory validation, three human NSCLC cell lines were selected: A549 (lung adenocarcinoma), H1975 (EGFR-mutant lung adenocarcinoma), and H520 (lung squamous cell carcinoma). All cell lines were procured from the American Type Culture Collection (ATCC) and cultured under standard conditions at 37°C with 5% CO_2_ in RPMI-1640 medium (Gibco, Thermo Fisher Scientific) supplemented with 10% fetal bovine serum (FBS) and 1% penicillin-streptomycin. These cell lines were selected based on their molecular characteristics: H1975 harbors the clinically relevant EGFR T790M mutation; A549 serves as a KRAS-mutant adenocarcinoma reference; and H520, although not typically ALK-positive, was included due to reports of low-level ALK mRNA expression in squamous NSCLC, allowing exploratory assessment of expression heterogeneity. Total RNA was extracted using the RNeasy Mini Kit (Qiagen, Germany), and RNA quality and concentration were evaluated using a NanoDrop 2000 spectrophotometer (Thermo Scientific) along with agarose gel electrophoresis to ensure integrity. Complementary DNA (cDNA) was synthesized from 1 µg of total RNA using the High-Capacity cDNA Reverse Transcription Kit (Applied Biosystems). Quantitative PCR (qPCR) was performed using PowerUp SYBR Green Master Mix (Applied Biosystems) and gene-specific primers targeting EGFR, ALK, KRAS, and PDCD1 (primer sequences and conditions are detailed in **Supplementary Table S3**). GAPDH was used as the endogenous reference gene. All reactions were carried out in triplicate on the QuantStudio 5 Real-Time PCR System (Applied Biosystems) under standard thermal cycling conditions: 95°C for 2 minutes, followed by 40 cycles of 95°C for 15 seconds and 60°C for 1 minute. Relative expression levels were calculated using the 2^(-ΔΔCt) method, using A549 cells as the baseline control. This integrated approach combining in silico data mining and cell line-based qPCR assays enabled a robust validation of biomarker expression profiles relevant to NSCLC.

## 3 Results

### 3.1 Structural study using SWISS-MODEL

Understanding the three-dimensional (3D) structures of critical biomarker proteins is essential for elucidating their functional roles in tumorigenesis, drug resistance, and immune evasion in lung cancer. Homology modeling offers a powerful tool when high-resolution crystallographic structures are unavailable. Using the SWISS-MODEL server, we generated accurate 3D models for EGFR, ALK, KRAS, and PD-1, which represent key oncogenic and immunomodulatory targets in non-small cell lung cancer (NSCLC).

#### 3.1.1 Protein selection

The four selected proteins were chosen based on their clinical importance in lung cancer, involvement in key signaling pathways, and their status as targets of approved therapies. Their sequences were retrieved from the UniProt database, and associated details are summarized in **Table 1**.

**Table 1:** Selected lung cancer biomarker proteins for homology modeling.

Protein	Role in Lung Cancer	Common Alterations	UniProt ID
Epidermal Growth Factor Receptor (EGFR)	Promotes proliferation via tyrosine kinase	L858R, exon 19 deletions, T790M	P00533
Anaplastic Lymphoma Kinase (ALK)	Oncogenic fusions and kinase activation	EML4–ALK, L1196M, G1269A	Q9UM73
Kirsten Rat Sarcoma Viral Oncogene (KRAS)	GTPase regulating MAPK and PI3K pathways	G12C, G12D, G12V	P01116
Programmed Cell Death Protein 1(PD-1)	Immune checkpoint; suppresses T-cell activity	PDCD1 upregulation or SNPs	Q15116

#### 3.1.2 Homology modeling workflow and quality assessment

Each amino acid sequence was submitted to SWISS-MODEL between January and April 2025. Template selection involved BLAST and HHblits searches against the SMTL (SWISS-MODEL Template Library). Models were built based on the best-fitting templates with optimal sequence identity, resolution, and coverage. Quality was assessed using: GMQE (Global Model Quality Estimation): a score between 0–1 indicating the expected accuracy. QMEAN Z-score: indicating the degree of similarity to high-resolution crystal structures (**Table 2**).

**Table 2:** Summary of homology modeling results using SWISS-MODEL.

Protein	Template PDB ID	Sequence identity (%)	GMQE	QMEAN Z-score	Model confidence
EGFR	7syd.1.C	~99.34%	0.62	–0.77	High (L858R, T790M captured)
ALK	3lct.1.A	~99.70%	0.15	–0.76	Moderate–High (DFG, ATP pocket resolved)
KRAS	4dso.1.A	~88.77%	0.86	–0.85	High (GTP-binding domain, G12C captured)
PD-1	7xad.4.A	~99.16%	0.72	–0.82	High (Ig-like fold, PD-L1 binding site resolved)

#### 3.1.3 Structural insights and ramachandran validation

The generated models revealed functional motifs and structural domains relevant to biomarker activity, mutation hotspots, and drug-binding interfaces. Quality assessment was further confirmed using MolProbity for stereochemical evaluation, including Ramachandran plots **Table 3**.

**Table 3:** Structural and mutational insights into modeled proteins.

Protein	Key structural features	Common mutations	Drug interaction sites	Therapeutic relevance	Model quality
Epidermal Growth Factor Receptor (EGFR)	N-lobe, C-lobe, hinge (ATP-binding cleft), αC-helix, A-loop	L858R (Exon 21), Exon 19 deletions, T790M	ATP-binding cleft, T790 site	Target of TKIs (e.g., gefitinib, Osimertinib); T790M causes steric hindrance	MolProbity: 1.36 Favored: 91.5% Outliers: 0.33%
Anaplastic Lymphoma Kinase (ALK)	Kinase fold, DFG motif, ATP-binding site, αC-helix	L1196M, G1269A, F1174L	Active site, ATP pocket	Target of ALK inhibitors (e.g., crizotinib, lorlatinib); mutations affect binding	MolProbity: 2.09 Favored: 93.14% Outliers: 1.96%
Kirsten Rat Sarcoma Viral Oncogene (KRAS)	G-domain (G1–G5 motifs), Switch I & II, P-loop	G12C, G13D, Q61H/L	P-loop near the allosteric pocket	Targeted by covalent inhibitors (e.g., sotorasib); mutations inhibit GTP hydrolysis	MolProbity: 1.38 Favored: 93.26% Outliers: 3.93%
Programmed Cell Death Protein 1(PD-1)	IgV-like domain, β-sandwich fold	PDCD1 polymorphisms (e.g., 7146G>A)	Ligand-binding domain (Y68, I126, E136)	Targeted by immune checkpoint inhibitors (e.g., nivolumab, pembrolizumab)	MolProbity: 0.67 Favored: 97.13% Outliers: 0.00%

**Figure 1:**
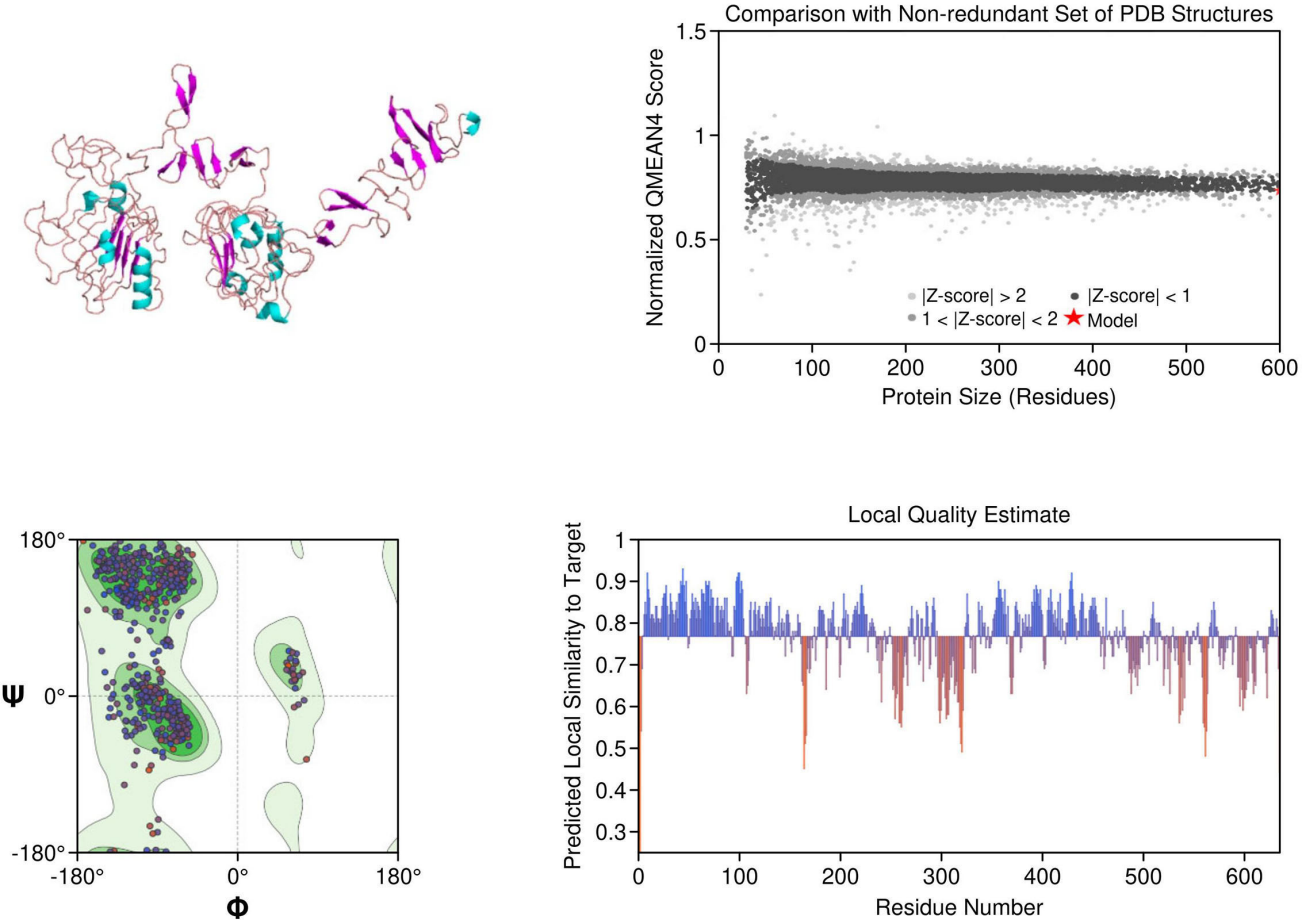
Homology model and validation of EGFR (P00533). The modeled structure of the Epidermal Growth Factor Receptor (EGFR) shows a well-defined kinase domain comprising the N-lobe, C-lobe, and ATP-binding cleft. Key regions such as the αC-helix, activation loop (A-loop), and the hinge region are visible. The structure supports the presence of clinically relevant mutations like L858R and T790M, which influence drug resistance. Ramachandran plot validation shows 91.5% residues in favored regions, with a MolProbity score of 1.36, confirming model quality.

EGFR: The structural model of EGFR provided detailed visualization of the kinase domain, including the N-lobe, C-lobe, hinge region, and ATP-binding cleft. Mutations L858R and T790M were positioned within the binding pocket, supporting known mechanisms of kinase activation and resistance to tyrosine kinase inhibitors (TKIs). The T790M substitution introduces steric hindrance, correlating with reduced inhibitor binding affinity. Ramachandran plot analysis revealed 91.5% residues in favored regions, with a low outlier rate, and a MolProbity score of 1.36, indicating high stereochemical quality (**Figure 1**).

**Figure 2:**
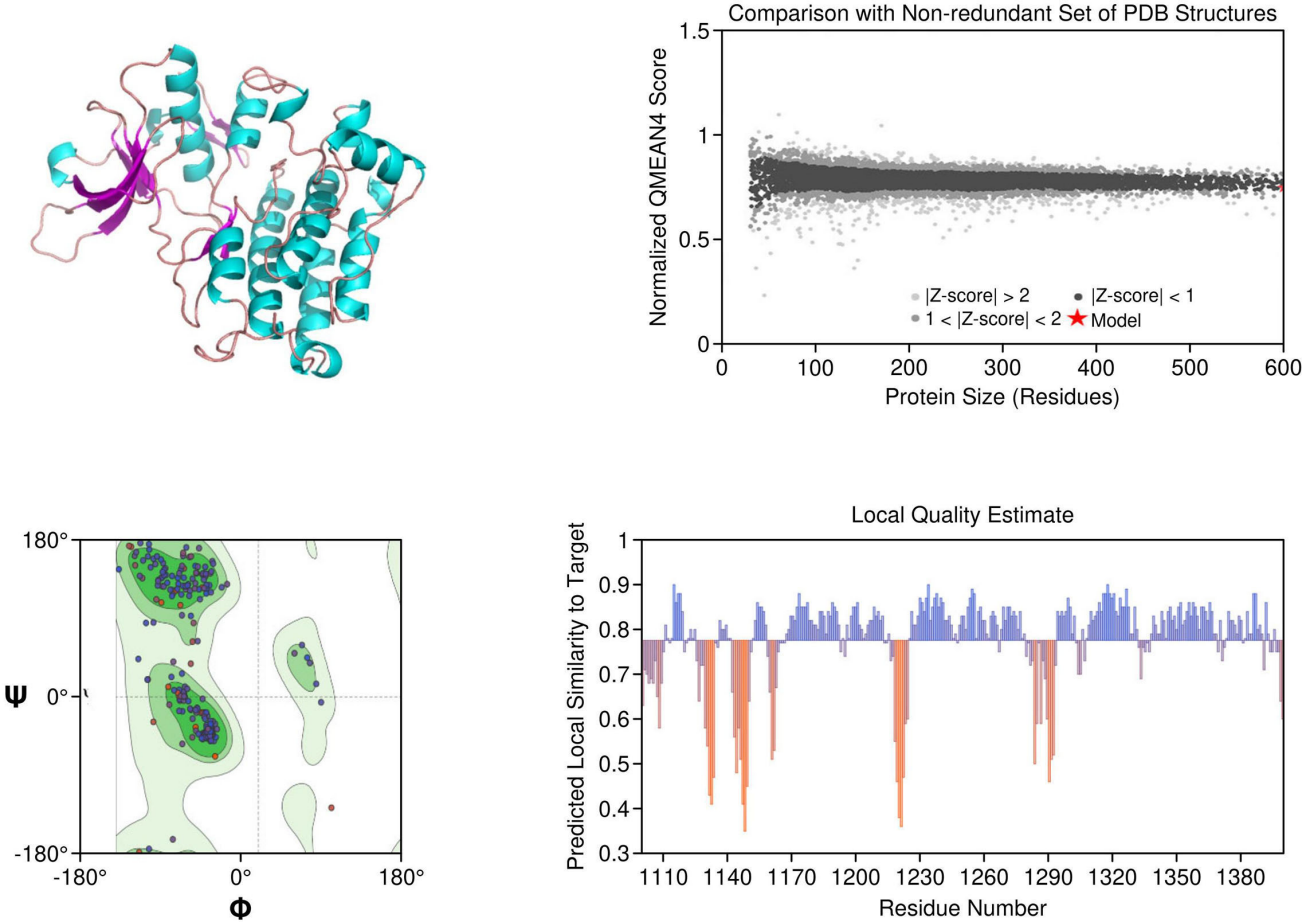
Homology model and validation of ALK (Q9UM73). The ALK model displays the canonical kinase fold, which includes the traditional ATP binding pocket, DFG motif, and αC-helix required for inhibitor binding. The locations of mutations associated with therapy resistance such as L1196M and F1174L can be mapped structurally. The model included 93.14% favored residues on the Ramachandran plot, and the MolProbity score was 2.09. The structural characteristics of the ALK model provide support for the model to be relevant for understanding aspects of ALK inhibitors with regard to design and resistance mechanisms.

ALK: The ALK model resolved key catalytic features, including the αC-helix, activation loop, and DFG motif. Resistance mutations L1196M and G1269A were located at or near the ATP-binding cleft, supporting their role in reducing drug efficacy. These structural positions validate why next-generation ALK inhibitors such as lorlatinib are effective in overcoming first-line resistance. The model achieved a MolProbity score of 2.09 with 93.14% residues in favored Ramachandran regions, indicating good overall geometry (**Figure 2**).

**Figure 3:**
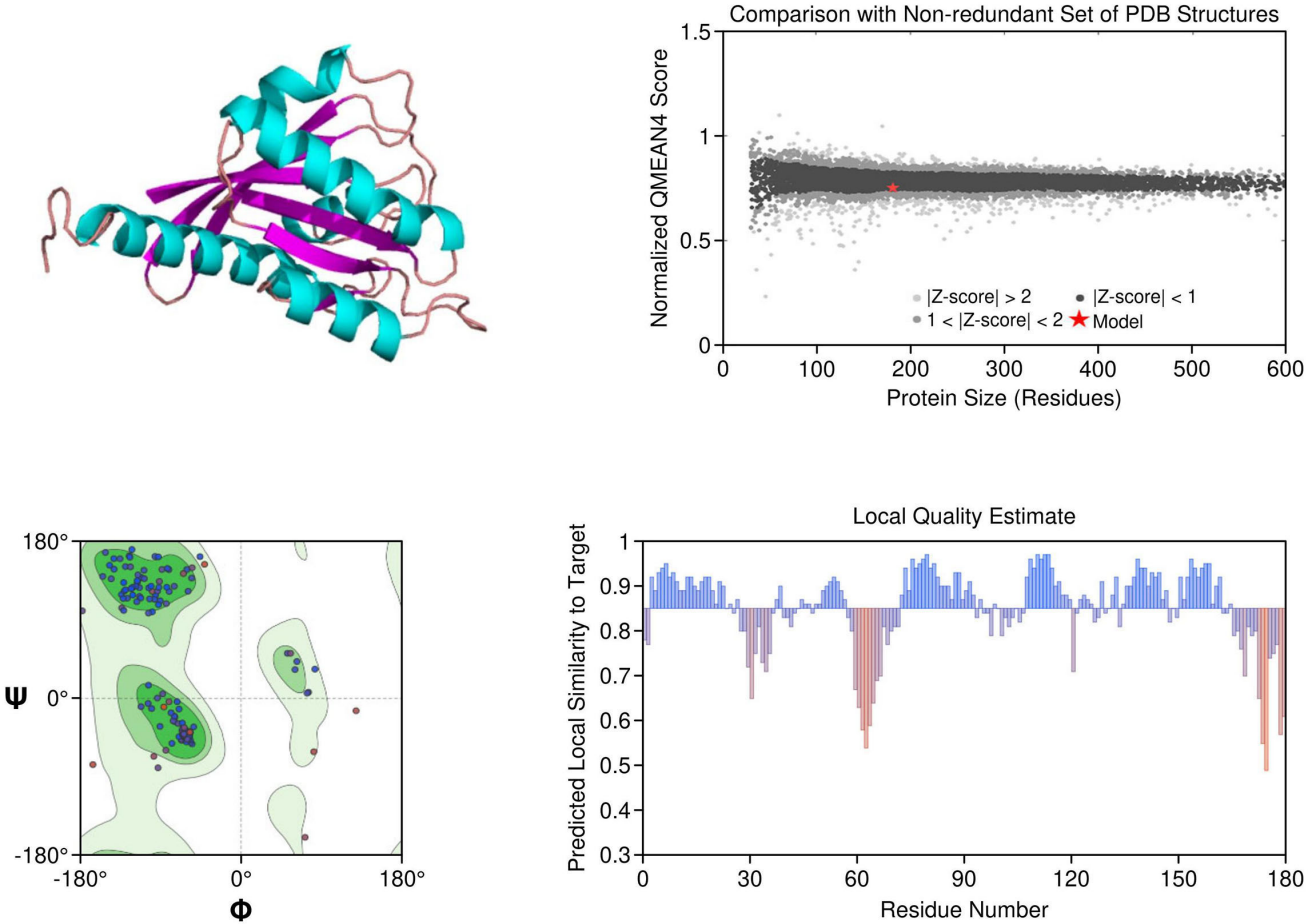
Homology model and validation of KRAS (P01116). KRAS structure reveals a compact G-domain with well-defined GTP-binding motifs (G1–G5) and flexible Switch I and II regions. The G12C mutation, located at a solvent-accessible site near the allosteric pocket, is visible and forms the basis for covalent inhibition strategies. Model validation with a MolProbity score of 1.38 and 93.26% favored residues on the Ramachandran plot confirms a reliable structure for drug-target interaction analysis.

KRAS: The homology model of KRAS revealed structurally distinct regions including the GTP-binding P-loop and the dynamic switch I/II regions. Mutation G12C was exposed in a solvent-accessible allosteric pocket, ideal for covalent targeting by inhibitors such as sotorasib. Other oncogenic hotspots, including G13D and Q61H, were also well resolved. The model had a MolProbity score of 1.38 and 93.26% of residues within favored regions, supporting its utility in in silico drug design (**Figure 3**).

**Figure 4:**
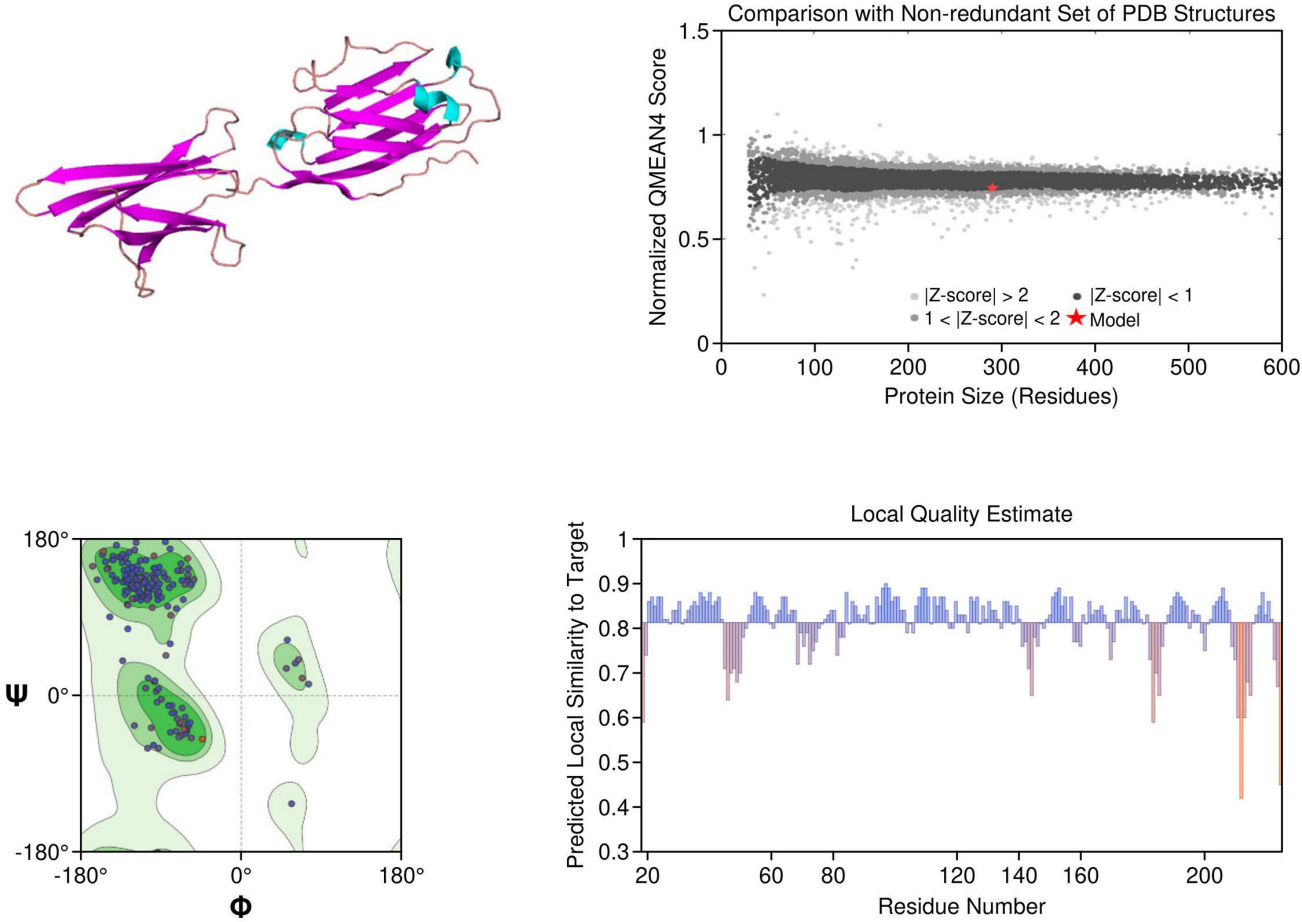
Homology model and validation of PD-1 (Q15116). The PD-1 homology model focuses on the IgV-like extracellular domain, which is a β-sandwich fold held together with disulfide bridges. The Y68, I126 and E136 residues involved in PD-L1 binding were all structurally defined in the homology model, this supports current checkpoint blockade approaches. Ramachandran analysis indicates high model quality, with 97.13% residues in favored regions and a low MolProbity score of 0.67, making it suitable for further immunotherapy-based structural studies.

PD-1: The PD-1 homology model exhibited a well-defined immunoglobulin variable (IgV)-like fold and included key residues involved in ligand binding, such as Tyr68, Ile126, and Glu136. Structural comparison with the crystallographic reference structure (PDB: 3RRQ) demonstrated a strong similarity, with a root-mean-square deviation (RMSD) of approximately 1.4 Å, confirming the model’s accuracy in capturing the IgV fold and the ligand-binding interface. These structural insights clarify binding interactions with therapeutic antibodies like nivolumab and pembrolizumab. Additionally, known PDCD1 polymorphisms relevant to immunotherapy response could be structurally localized. The model showed the highest stereochemical quality, with a MolProbity score of 0.67 and 97.13% favored residues (**Figure 4** **).**

### 3.2 STRING interaction network analysis

**Figure 5:**
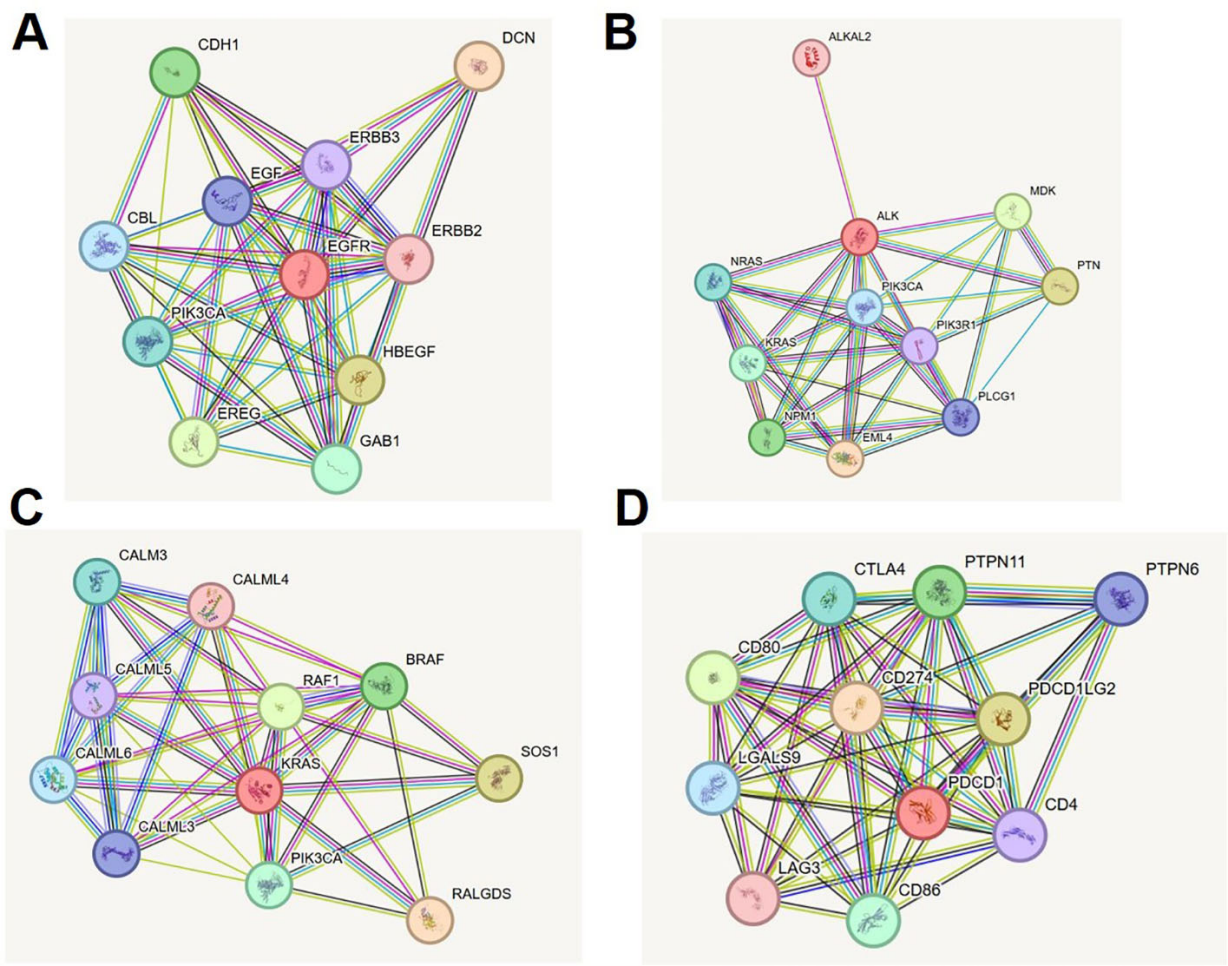
STRING protein-protein interaction networks of lung cancer biomarkers. **(A)** EGFR interaction network showing key ligands, co-receptors, and downstream signaling molecules (MAPK, PI3K/AKT, JAK/STAT). **(B)** ALK interaction network illustrating fusion partners, growth factors, and effector proteins in oncogenic cascades. **(C)** KRAS network with upstream activators, MAPK and PI3K effectors, and calcium-modulating regulators. **(D)** PD-1 network displaying immune checkpoint ligands, co-stimulatory molecules, phosphatases, and co-inhibitory regulators.

The STRING protein–protein interaction (PPI) analysis of EGFR, ALK, KRAS, and PD-1 revealed a complex, yet functionally coordinated molecular landscape in lung cancer. EGFR emerged as a central receptor tyrosine kinase, forming high-confidence interactions (score = 0.999) with ligands HBEGF, EREG, and EGF, as well as co-receptors ERBB2 and ERBB3. These interactions propagate downstream signaling through canonical MAPK, PI3K/AKT, and JAK/STAT pathways, while intracellular adaptors like PIK3CA, CBL, and GAB1 support signaling amplification, endocytosis, and feedback regulation (**Supplementary Table S4**, **Figure 5A**). In parallel, ALK demonstrated interactions with its oncogenic fusion partner EML4, high-affinity ligands PTN and MDK, and signaling mediators such as PIK3R1, PLCG1, KRAS, and NRAS. These nodes anchor ALK within PI3K-AKT and MAPK cascades. Notably, ALKAL2 was also identified as a ligand for ALK, reinforcing its receptor-ligand activation loop (**Supplementary Table S5**, **Figure 5B**). KRAS exhibited a tightly regulated network comprising upstream activators (SOS1, RALGDS), MAPK effectors (RAF1, BRAF), and PI3K axis mediators (PIK3CA). Intriguingly, interaction with calcium-modulating proteins from the calmodulin family (CALML3–6) revealed an additional layer of regulatory complexity, highlighting calcium-mediated modulation of oncogenic signaling (**Supplementary Table S6**, **Figure 5C**). Finally, PD-1 (PDCD1) was shown to interface with ligands CD274 (PD-L1) and PDCD1LG2 (PD-L2), which mediate immune checkpoint suppression. The receptor also interacted with co-stimulatory proteins CD80 and CD86 and with intracellular phosphatases SHP2 (PTPN11) and SHP1 (PTPN6), which inhibit TCR pathway components. Additional interactions with CTLA4, LAG3, LGALS9, and CD4 placed PD-1 at the center of a dynamic immunoregulatory network critical for tumor immune evasion (**Supplementary Table S7**, **Figure 5D**). These comprehensive STRING networks reinforce the multifaceted signaling and regulatory roles of these key biomarkers in lung cancer and provide mechanistic insight into their therapeutic potential. The multifaceted signaling and regulatory roles of these key biomarkers in lung cancer and provide mechanistic insight into their value in precision medicine.

### 3.3 Molecular docking interpretation

**Figure 6:**
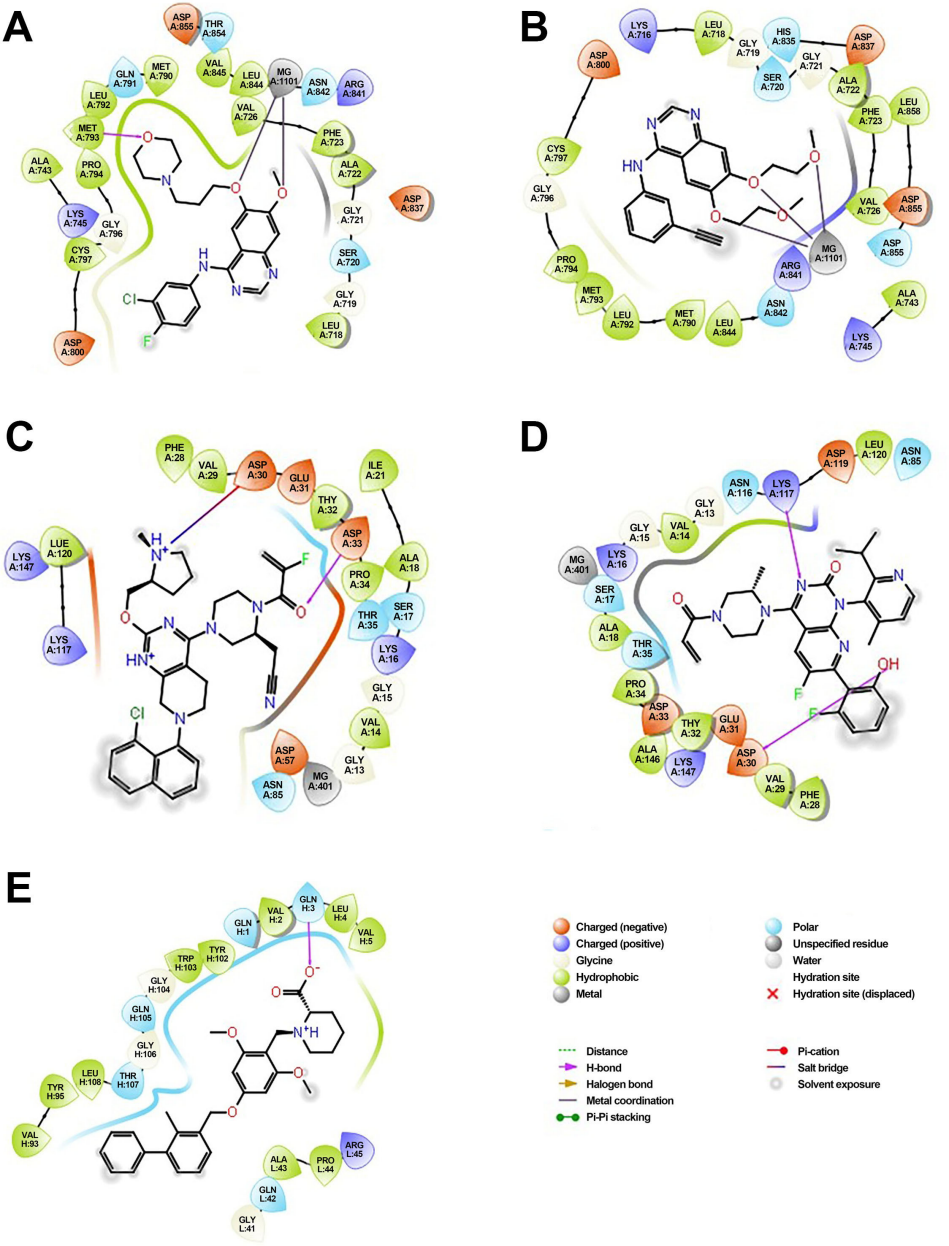
Molecular docking of small-molecule inhibitors with EGFR, KRAS, and PD-L1: predicted binding poses and key residue interactions. **(A)** Predicted binding pose of Gefitinib within the ATP-binding pocket of EGFR, showing a strong hydrogen bond with MET793 and hydrophobic interactions with LEU718 and VAL726. **(B)** Binding conformation of Erlotinib in EGFR, forming hydrogen bonds with MET793 and ASP800 and hydrophobic contacts with ALA743. **(C)** Adagrasib docked in the KRAS G12C switch II pocket, forming halogen and Pi–cation interactions with TYR32 and PHE28 and a hydrogen bond with ASP33. **(D)** Sotorasib interacting with the KRAS switch II pocket, displaying Pi–Pi stacking with PHE28 and hydrogen bonds with SER17 and ASP30. **(E)** PD-L1 ligand binding to the PD-L1 receptor groove, forming hydrogen bonds with GLN104, GLN114, and GLN115 and hydrophobic contacts with VAL93, LEU108, and TYR102.

The molecular docking analysis revealed that each inhibitor displayed distinct binding affinities and interaction profiles with its respective target, as summarized in Table 9. Among all compounds, Gefitinib exhibited the strongest binding to EGFR, with a binding energy of −5.94 kcal/mol and a dissociation constant (Kd) of 4.38 × 10^-5^ M. Its high affinity was supported by a strong hydrogen bond with MET793 and extensive hydrophobic interactions with LEU718 and VAL726 within the ATP-binding pocket of EGFR (**Figure 6A**), stabilizing the ligand effectively. Erlotinib, another EGFR-targeting inhibitor, also demonstrated high binding affinity (−5.78 kcal/mol, Kd = 5.73 × 10^-5^ M), forming critical interactions with MET793 and ASP800, alongside hydrophobic contacts involving ALA743 (**Figure 6B**). Its slightly lower binding energy compared to Gefitinib suggests comparable but marginally reduced stability in the receptor pocket. As shown in **Table 4**, its ligand efficiency of 0.19 suggests high pharmacological relevance. The KRAS G12C inhibitors, Adagrasib and Sotorasib, showed moderate binding energies (−3.94 and −3.72 kcal/mol, respectively) and lower affinity compared to EGFR inhibitors. However, both ligands displayed target-specific interactions, supporting their selective binding. Adagrasib (**Figure 6C**) formed halogen and Pi–cation interactions with TYR32 and PHE28, as well as a hydrogen bond with ASP33, whereas Sotorasib (**Figure 6D**) targeted the KRAS switch II pocket through Pi–Pi stacking with PHE28 and hydrogen bonds with SER17 and ASP30. These interactions, reflected in ligand efficiencies ranging from 0.17 to 0.20 (**Table 4** **),** support their efficacy despite modest binding energy. To support reproducibility, the small-molecule ligands used in this study were annotated with public database identifiers. Gefitinib, used as an EGFR inhibitor, corresponds to PubChem CID: 123631. For the KRAS G12C-targeted docking studies, sotorasib and adagrasib were modeled based on structures closely matching PubChem CIDs 134557218 and 137517260, respectively. The PD-L1 small-molecule inhibitor evaluated in this study shares structural similarity with known checkpoint antagonists such as those listed under PubChem CIDs 91663303 and 168679817. These identifiers allow independent retrieval of compound structures for future replication and further in silico optimization. The PD-L1 ligand demonstrated a robust binding energy of −5.54 kcal/mol and Kd of 8.60 × 10^-5^ M, underpinned by three hydrogen bonds with GLN104, GLN114, and GLN115, as well as hydrophobic contacts with VAL93, LEU108, and TYR102 (**Figure 6E**). These interactions suggest its potential to disrupt PD-1/PD-L1 signaling. The PD-L1 ligand used in docking is a known small-molecule antagonist (PubChem CID: 132194007) reported to disrupt PD-1/PD-L1 binding *in-vitro.* In contrast, Crizotinib and Alectinib, both ALK inhibitors, yielded weaker docking results with higher Kd values (1.8 × 10^-^³ and 5.61 × 10^-4^ M, respectively) and lower ligand efficiencies (**Table 4**). Though clinically effective, their modeled binding energies (−3.75 and −4.43 kcal/mol) indicate suboptimal binding stability in this computational context. Overall, **Table 4** provides a comparative overview of the binding performance and efficiencies, highlighting Gefitinib, Erlotinib, and the PD-L1 ligand as the most promising candidates, with KRAS inhibitors showing moderate but target-specific activity, and ALK inhibitors underperforming in this model.

**Table 4:** Binding affinity scores (ΔG in kcal/mol) of top docked models for lung cancer biomarkers with their respective ligands.

Compound	Structure	Binding energy	Kd	Ligand efficiency
Gefitinib	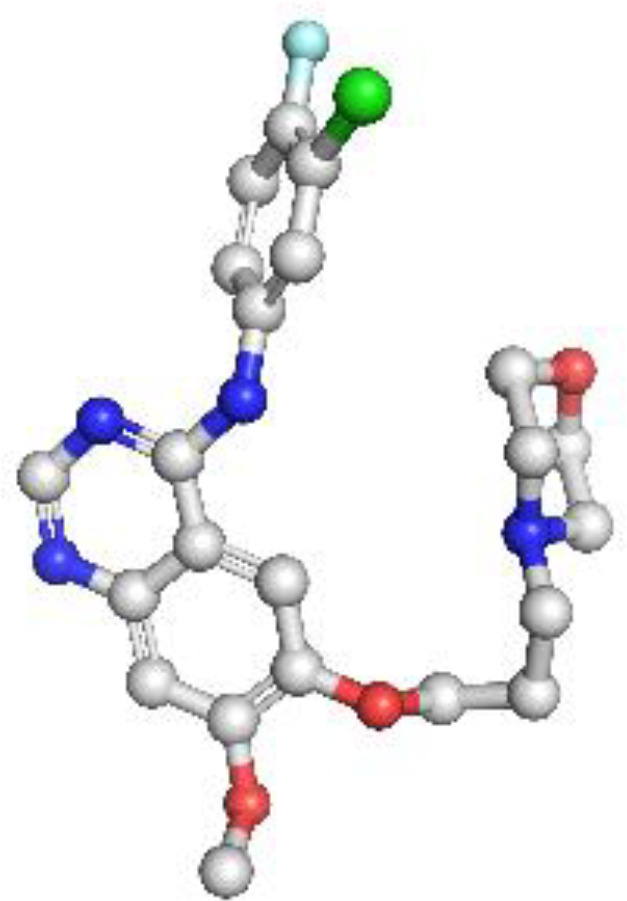	-5.94	4.38x10^-5^	0.17
Erlotinib	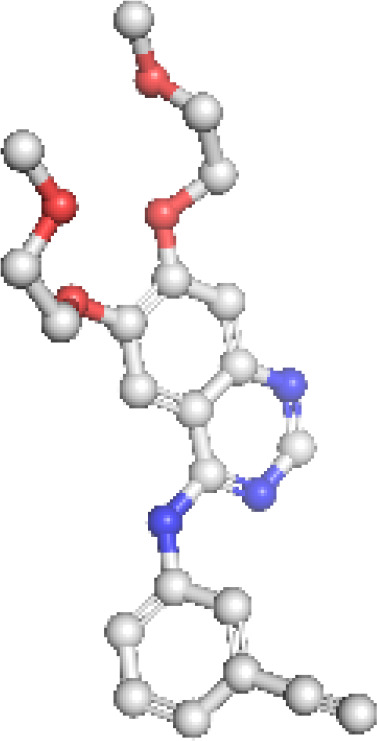	-5.78	5.73x10^-5^	0.19
Crizotinib	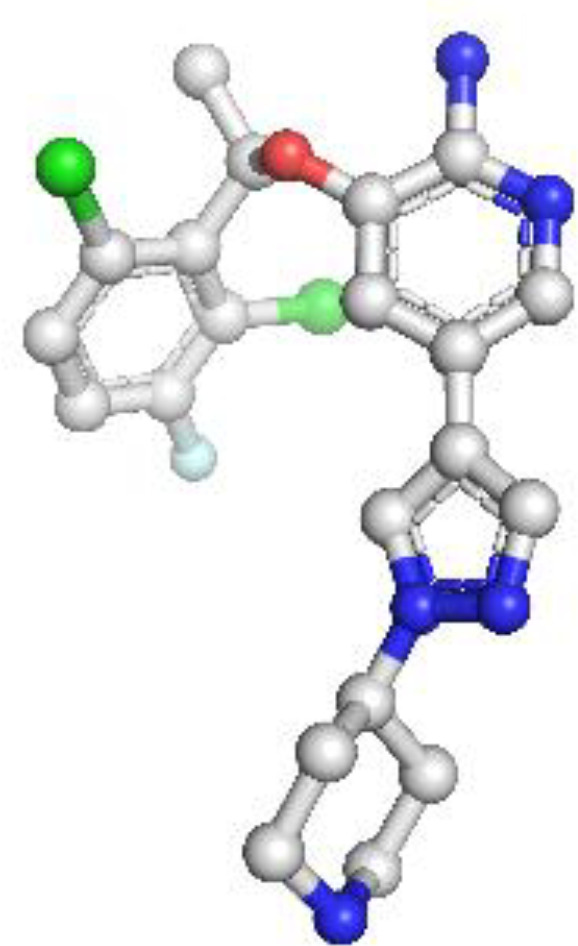	-3.75	1.8x10^-3^	0.14
Alectinib	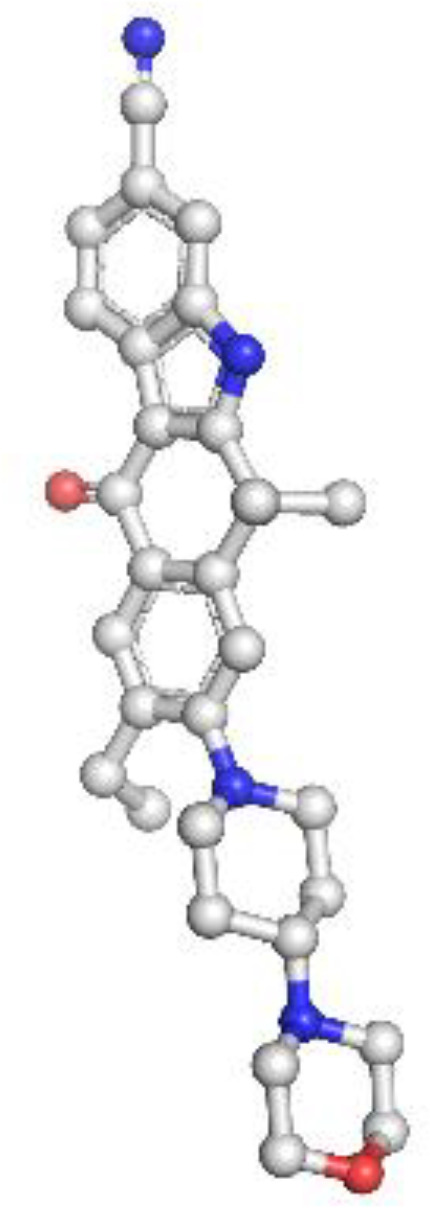	-4.43	5.61x10^-4^	0.13
Sotorasib	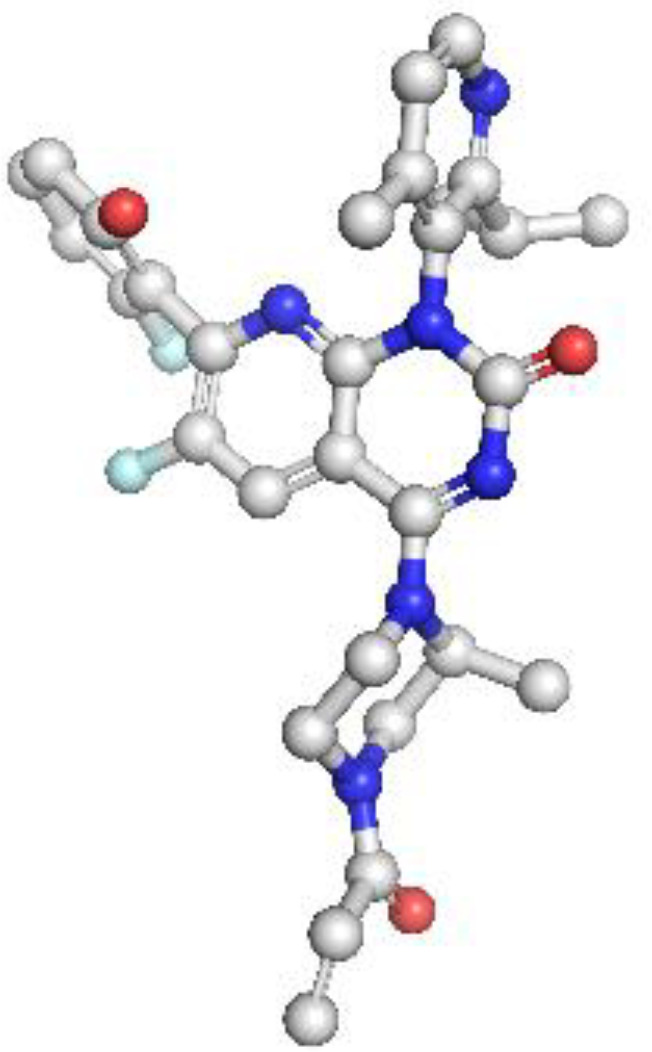	-3.72	1.86x10^-3^	0.20
Adagrasib	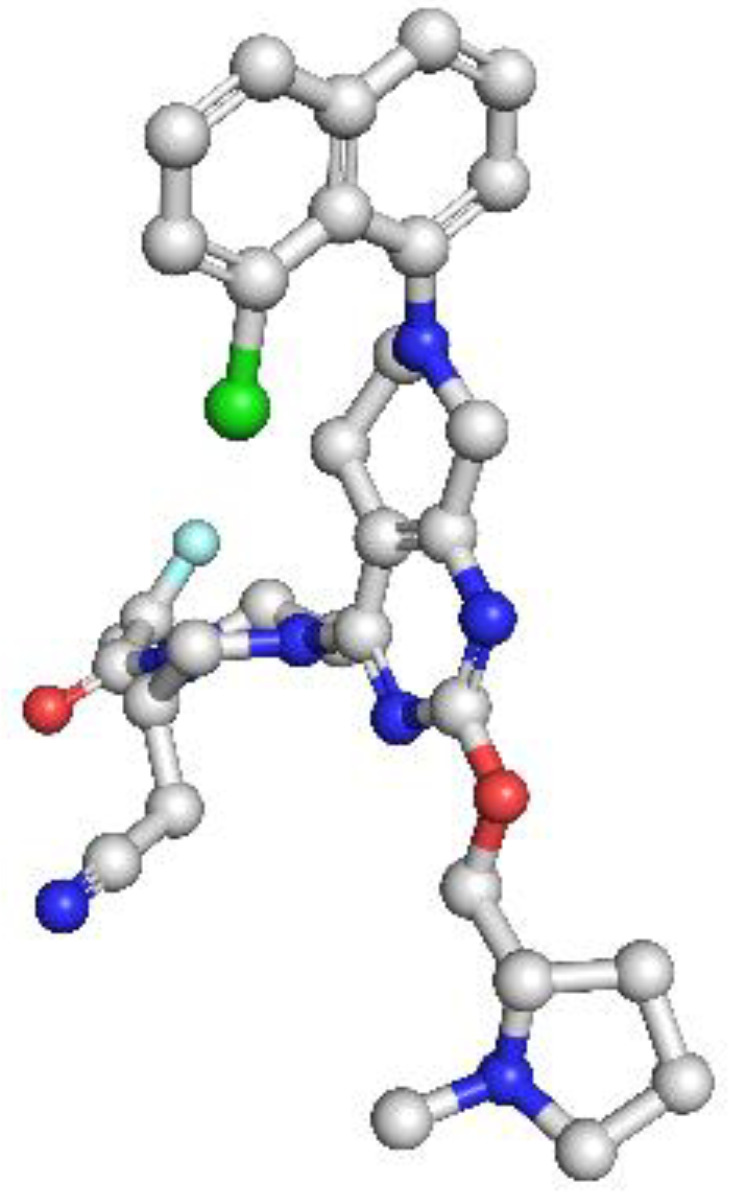	-3.94	1.28x10^-3^	0.17
PD L1	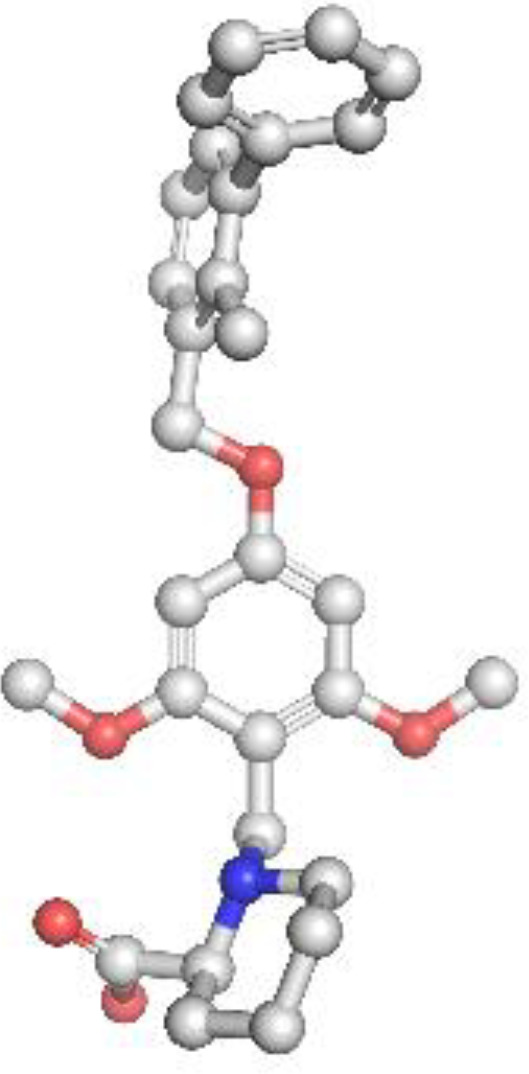	-5.54	8.60x10^-5^	0.20

### 3.4 Transcriptomic validation of key biomarkers

The selected biomarkers (EGFR, ALK, KRAS, and PDCD1), demonstrated significantly higher expression in NSCLC tumor tissues compared to adjacent normal tissues (p < 0.01). In both LUAD and LUSC cohorts, EGFR and KRAS showed consistently elevated expression. ALK was upregulated in a subset of tumors, particularly in non-smokers, while PDCD1 was predominantly expressed in immune-rich tumor microenvironments. UALCAN analysis further revealed stage-specific expression patterns for all four genes. Promoter methylation data indicated low methylation levels for EGFR, ALK, and KRAS, suggesting that their overexpression is driven by transcriptional activation rather than epigenetic silencing. To experimentally validate these findings, quantitative PCR (qPCR) was conducted on three human NSCLC cell lines: A549, H1975, and H520. Relative expression levels were normalized to GAPDH and calculated using the 2^(-ΔΔCt) method, with A549 serving as the reference baseline. EGFR expression was markedly elevated in H1975 cells, consistent with its EGFR-mutant status. ALK showed increased expression in H520, while KRAS expression was higher in H1975 compared to A549 and H520. PDCD1 was modestly expressed across all three lines, with the highest levels detected in H520 cells (**Table 5**). These laboratory findings closely mirrored the in silico transcriptomic trends and further substantiated the relevance of these genes in NSCLC biology.

**Table 5:** Transcriptomic and expression levels validation of key NSCLC biomarkers.

Gene	*In Silico* differential expression (Tumor vs. Normal)	Stage-specific pattern (UALCAN)	Promoter methylation (UALCAN)	qPCR expression in NSCLC cell lines<br>(Relative to A549, 2^−ΔΔCt)
*EGFR*	↑ LUAD, ↑ LUSC (p < 0.01)	↑ in advanced stages	Low methylation	H1975: ↑↑
				H520: ↑
*ALK*	↑ Subset of LUAD (p < 0.01)	↑ in early LUAD stages	Low methylation	H1975: ↑
				H520: ↑↑
*KRAS*	↑ LUAD > LUSC (p < 0.01)	Consistently high across stages	Low methylation	H1975: ↑↑
				H520: ↑
*PDCD1*	↑ in immune-rich LUAD/LUSC (p < 0.01)	↑ with tumor stage	Mild promoter methylation	H1975: ↑
				H520: ↑↑

LUAD – Lung adenocarcinoma; LUSC – Lung squamous cell carcinoma; ↑ – moderate increase; ↑↑ – strong increase.

## 4 Discussion

Lung cancer remains one of the most lethal malignancies worldwide, with non-small cell lung cancer (NSCLC) accounting for nearly 85% of all cases. Late-stage diagnosis and therapeutic resistance significantly hinder clinical outcomes (20, 21). In this study, we applied an integrated multi-omics approach combining structural modeling, protein–protein interaction (PPI) network analysis, and both *in-silico* and experimental transcriptomic validation to investigate four clinically actionable biomarkers—EGFR, ALK, KRAS, and PDCD1—central to NSCLC pathogenesis and therapy.

Homology models constructed via SWISS-MODEL demonstrated high stereochemical integrity and structural accuracy, capturing key oncogenic and resistance-conferring mutations, including EGFR T790M, ALK L1196M, and KRAS G12C. These mutations are well-documented contributors to resistance against tyrosine kinase inhibitors (TKIs), either by altering the ATP-binding pocket geometry or introducing steric hindrance (11, 20, 22). Our models provided atomic-level insight into these conformational changes, facilitating rational drug design strategies for TKI-resistant NSCLC subtypes. Notably, EGFR structural domains associated with ligand-binding and dimerization were well-resolved, reinforcing its importance in receptor tyrosine kinase inhibition. The ALK-kinase domain model aligned with oncogenic ALK-fusion conformations seen in EML4–ALK, while KRAS exhibited a structurally conserved G-domain required for GTP/GDP cycling—an established therapeutic vulnerability. The PDCD1 (PD-1) model further revealed its immunoglobulin-like extracellular topology, which is critical for interactions with checkpoint ligands such as PD-L1 and PD-L2 (23). Importantly, the homology model of PD-1 provides detailed spatial insights into the IgV-like domain and key binding residues such as Y68, I126, and E136, which are instrumental in guiding the rational design of nanocarrier-bound checkpoint inhibitors. These structural features can be harnessed to engineer nanoparticle-conjugated anti-PD-1 antibodies or ligands that enhance targeted delivery and immune activation in the tumor microenvironment (24).

To elucidate the functional context of these biomarkers, we employed STRING-based PPI network analysis. EGFR and ALK were located upstream in key oncogenic pathways, interacting with adaptors such as GRB2, SHC1, and ERBB2, indicating canonical MAPK and PI3K/AKT activation. The KRAS interactome prominently featured RAF1, SOS1, and BRAF, corroborating its centrality in RAS–RAF–MEK–ERK signaling cascades (25, 26). Interestingly, KRAS also exhibited novel associations with calmodulin-like proteins (CALM3, CALML6), suggesting potential modulation by calcium signaling. PDCD1 formed inhibitory networks with CD80/86 and SHP2, aligning with checkpoint inhibition and immune evasion frameworks (27, 28). These network-level interactions reinforced our structural data and provided a dual-dimensional insight into the biological roles and therapeutic tractability of these proteins.

Transcriptomic validation further strengthened the evidence for biomarker relevance. In silico expression analysis using GEPIA2, TNMplot, and UALCAN revealed significantly elevated expression of EGFR, ALK, and KRAS in NSCLC tissues versus normal lung tissues (p < 0.01), while PDCD1 was selectively upregulated in tumors with high immune infiltration, consistent with its immunomodulatory function (29, 30). Stage-specific analysis showed increasing PDCD1 expression with advancing tumor stages, suggesting its role as a dynamic immunological biomarker. Promoter methylation profiles indicated low methylation in EGFR, ALK, and KRAS, suggesting transcriptional activation rather than epigenetic silencing. To validate these findings experimentally, we conducted qPCR-based expression profiling in NSCLC cell lines—A549, H1975, and H520. The EGFR transcript was significantly upregulated in H1975, a cell line harboring the T790M mutation, consistent with clinical resistance phenotypes (31). ALK expression was elevated in H520, aligning with subtype-specific expression trends. KRAS showed robust expression in both H1975 and H520, reflecting its ubiquitous role across NSCLC subtypes, particularly in smokers (32). Notably, PDCD1 expression was highest in H520, despite overall lower levels compared to the other biomarkers, reinforcing its immune-context-specific expression and its utility in checkpoint inhibitor studies. Notably, KRAS interactions with calmodulin-like proteins (CALML3–6) suggest a noncanonical regulatory axis that may involve calcium-dependent modulation of RAS activity. This interaction could influence KRAS-driven oncogenesis through alterations in MAPK signaling sensitivity, a mechanism that warrants further investigation in the context of calcium signaling dynamics and therapeutic resistance.

These multi-layered analyses—spanning structural, functional, and expression dimensions—offer a comprehensive biomolecular characterization of four pivotal NSCLC biomarkers. Unlike previous studies focusing on isolated data types or single gene validation, our approach bridges computational modeling, systems biology, and laboratory experimentation to present a more integrated view. The combination of 3D modeling, molecular docking, and expression correlation enables mechanistic insights into resistance mutations, network dependencies, and therapeutic vulnerability. Docked poses were validated by comparing them to crystallographic reference structures, yielding RMSD values ≤ 2.1 Å, which confirms the reliability of the predicted binding conformations. For example, the high docking affinity of gefitinib with the EGFR model aligns well with its established clinical potency, whereas the comparatively weaker binding observed in the PD-1 model reflects a more nuanced interaction profile, consistent with the behavior of immunomodulatory agents. These insights pave the way for nanomedicine-based translation, especially the use of mesoporous silica nanoparticles, liposomes, or lipid-based nanocarriers for PD-1-targeted immune checkpoint blockade, with enhanced tumor specificity and reduced off-target effects (33). Additionally, the high-affinity binding of gefitinib to the EGFR kinase domain and the validated structural model of KRAS G12C support the development of nanocarrier-mediated delivery systems for these TKIs. Encapsulating gefitinib, sotorasib, or adagrasib within polymeric nanoparticles or lipid bilayered vesicles may enhance drug stability, improve tumor targeting via enhanced permeability and retention (EPR) effect, and overcome solubility-related pharmacokinetic limitations (34).

Building on the strong foundation of *in-silico* and cell line-based validations presented here, future studies will expand to include patient-derived tumor samples to better capture the heterogeneity and microenvironmental complexity of NSCLC. Incorporating proteomic analyses and functional assays will also enhance understanding of biomarker expression at the protein and pathway activity levels. Although not directly analyzed in this study, DepMap and CCLE resources are earmarked for future validation of therapeutic responses linked to our biomarker panel. Further exploration of PD-1-targeted nanosystems—such as antibody-functionalized nanogels or pH-sensitive micelles—could yield translational advances in nano-immunotherapy. To further improve structural insights, we plan to leverage emerging AI-driven tools like Alpha Fold_2_ and molecular dynamics simulations for more precise modeling of mutant proteins. While our current study does not simulate nanomaterial–biomarker interactions, future work will incorporate nanocarrier conjugation studies, leveraging docking and molecular dynamics tools to evaluate binding specificity and release kinetics. Together, these approaches will deepen the translational relevance of biomarker characterization and support the development of more effective targeted therapies and nanomaterials-based immunotherapies for NSCLC patients.

## 5 Conclusion

This study comprehensively characterized four key NSCLC biomarkers—EGFR, ALK, KRAS, and PDCD1—through an integrated framework combining structural modeling, protein–protein interaction analysis, and transcriptomic validation using public datasets and experimental qPCR in cell lines. Our findings confirmed their significant overexpression and pivotal roles in oncogenic signaling and immune regulation, reinforcing their value as diagnostic and therapeutic targets in lung cancer. The structural elucidation of PDCD1 (PD-1) offers a mechanistic basis for designing nanomaterial-assisted immune checkpoint interventions. The conformational insights into EGFR, ALK, and KRAS further support the development of nanocarrier-based delivery systems for targeted tyrosine kinase or GTPase inhibition. By aligning molecular structure with functional data, this work sets the stage for the rational design of nanomaterials that enhance drug delivery, improve immune modulation, and overcome resistance mechanisms. This multidisciplinary study not only validates established molecular targets but also bridges structural biology with emerging nano-immunotherapy strategies. These results provide a solid foundation for translational research involving patient-derived tumor models and nanotechnology-driven interventions aimed at advancing precision oncology in NSCLC.

## Data Availability

The datasets presented in this study can be found in online repositories. The names of the repository/repositories and accession number(s) can be found in the article/**Supplementary Material**.
