# Supplementary material for: Integrative profiling of lung cancer biomarkers EGFR, ALK, KRAS, and PD-1 with emphasis on nanomaterials-assisted immunomodulation and targeted therapy
Source: Front Immunol. 2025 Aug 25;16:1649445. doi: 10.3389/fimmu.2025.1649445 (PMC12414981; doi:10.3389/fimmu.2025.1649445)
Supplement: Supplementary file 1 [file Table1.docx]

**Title: Integrative Profiling of Lung Cancer Biomarkers EGFR, ALK, KRAS, 1 and PD-1 with Emphasis on Nanomaterials-Assisted 2 Immunomodulation and Targeted Therapy**

**Table S1: Common biomarkers in non-small cell lung cancer (NSCLC), their molecular types, functions, clinical relevance, and current targeted therapies.**

| **Biomarker** | **Gene** | **Type** | **Function** | **Clinical Relevance** | **Targeted Therapies** |
| --- | --- | --- | --- | --- | --- |
| **EGFR** (Epidermal Growth Factor Receptor) | *EGFR* | Activating mutations (e.g., L858R, exon 19 deletions) | Receptor tyrosine kinase promotes cell proliferation and survival | Common in NSCLC, especially in non-smokers and Asian populations; mutations confer sensitivity to TKIs | Gefitinib, Erlotinib, Afatinib, Osimertinib |
| **ALK** (Anaplastic Lymphoma Kinase) | *ALK* | Gene fusion (e.g., EML4–ALK) | Constitutive kinase activity due to fusion with EML4 drives oncogenesis | Found in ~5% of NSCLC, especially in younger non-smokers | Crizotinib, Alectinib, Ceritinib, Lorlatinib |
| **KRAS** (Kirsten Rat Sarcoma Viral Oncogene) | *KRAS* | Point mutations (e.g., G12C) | Small GTPase; promotes downstream signalling via MAPK and PI3K pathways | Occurs in ~25–30% of NSCLC; G12C is now targetable | Sotorasib, Adagrasib |
| **PD-1**  (Programmed Cell Death Protein 1 ) | *PDCD1*, *CD274* | Overexpression / immune checkpoint activation | Suppresses T-cell activity; allows tumours to evade immune response | Predicts response to checkpoint inhibitor therapy; overexpressed in many NSCLCs | Nivolumab, Pembrolizumab, Atezolizumab |
| **ROS1** (ROS Proto-Oncogene 1) | *ROS1* | Gene fusion (e.g., CD74–ROS1) | Receptor tyrosine kinase; fusion leads to constitutive activation | Found in 1–2% of NSCLC, typically in never-smokers | Crizotinib, Entrectinib |
| **RET** (Rearranged During Transfection) | *RET* | Gene fusion (e.g., KIF5B–RET) | Receptor tyrosine kinase; fusion drives oncogenic signalling | Occurs in ~1–2% of NSCLC, is more frequent in younger patients | Selpercatinib, Pralsetinib |
| **MET** (Mesenchymal-Epithelial Transition Factor) | *MET* | Exon 14 skipping mutation, amplification | Receptor tyrosine kinase; loss of degradation leads to sustained signalling | Present in ~3–4% of NSCLC; associated with aggressive tumour behaviour | Capmatinib, Tepotinib |
| **BRAF** (B-Raf Proto-Oncogene) | *BRAF* | V600E point mutation | Serine/threonine kinase in the MAPK pathway; promotes cell growth | Occurs in ~1–3% of NSCLC; V600E is actionable | Dabrafenib + Trametinib |

**Table S2: Supplementary Table S1. Transcriptomic Expression Validation of Lung Cancer Biomarkers (qPCR-style summary)**

| **Gene** | **Database Used** | **Tissue Comparison** | **Log^₂^ Fold Change** | ***p*-value** | **Expression Pattern** | **Clinical Association** |
| --- | --- | --- | --- | --- | --- | --- |
| EGFR | GEPIA2 | LUAD vs. Normal | 2.1 | <0.001 | Upregulated | High in LUAD; associated with stage II–III |
|  | TNMplot | NSCLC vs. Normal (RNA-seq) | 1.95 | <0.001 | Upregulated | Consistent across array/RNA-seq |
|  | UALCAN | LUAD Stage I–IV | Progressive ↑ | <0.01 | Stage-dependent | Elevated in advanced stages |
| ALK | GEPIA2 | LUAD vs. Normal | 1.56 | <0.01 | Upregulated | Higher in younger, non-smokers |
|  | TNMplot | NSCLC vs. Normal (RNA-seq) | 1.42 | <0.01 | Upregulated | Stronger in LUAD than LUSC |
|  | UALCAN | LUAD Stage I–IV | Mild ↑ in II–IV | <0.05 | Stage-associated | Therapeutic relevance increases with fusion |
| KRAS | GEPIA2 | LUAD vs. Normal | 2.38 | <0.001 | Strongly Upregulated | Associated with G12C mutation frequency |
|  | TNMplot | NSCLC vs. Normal (array) | 2.2 | <0.001 | Upregulated | High in smokers |
|  | UALCAN | LUAD Stage I–IV | Sustained ↑ | <0.01 | Consistent | High expression in all stages |
| PDCD1 | GEPIA2 | LUAD vs. Normal | 1.75 | <0.01 | Upregulated | Linked to inflamed tumour microenvironment |
|  | TNMplot | NSCLC vs. Normal (RNA-seq) | 1.61 | <0.01 | Upregulated | Predictive of ICI response |
|  | UALCAN | LUAD Stage I–IV | Peak in Stage III | <0.05 | Immune-related ↑ | Correlates with PD-L1 expression |

**Table S3. Primer Sequences and qPCR Conditions Used for Biomarker Validation**

| **Gene** | **Forward Primer (5'→3')** | **Reverse Primer (5'→3')** | **Amplicon Size (bp)** | **Annealing Temp (°C)** | **Reference or Design Tool** |
| --- | --- | --- | --- | --- | --- |
| **EGFR** | AGGCACGAGTAACAAGCTCAC | ATGAGGACATAACCAGCCACC | 120 | 60°C | Primer-BLAST (NCBI) |
| **ALK** | TTCTACACCAGTGGCTGGTGA | CCTTGCAGTGTAGTGGCTTCT | 135 | 60°C | Primer3Plus |
| **KRAS** | TGTGGTAGTTGGAGCTGGTG | CGTAGGCAAGAGACAGGTTTCT | 142 | 60°C | Primer-BLAST (NCBI) |
| **PDCD1** | TCCAGGATGGTTCTTAGACTCCC | GGTGTTGTTGATCTGGGCCTTC | 118 | 60°C | Primer3Plus |
| **GAPDH** | GAAGGTGAAGGTCGGAGTCA | GACAAGCTTCCCGTTCTCAG | 101 | 60°C | Endogenous control |

**Table S4: Detailed Overview of Predicted EGFR Functional Partners**

| **Protein** | **Full Name** | **Function/Role** | **Mechanism of Interaction** | **Pathways Involved** | **Confidence Score** |
| --- | --- | --- | --- | --- | --- |
| \| **EGFR** \| \| --- \| | Epidermal Growth Factor Receptor | Receptor tyrosine kinase that binds EGF ligands and activates MAPK, PI3K/AKT, and JAK/STAT pathways | Ligand-induced dimerisation and autophosphorylation | MAPK, PI3K/AKT, JAK/STAT | 0.999 |
| \| **DCN** \| \| --- \| | Decorin | Extracellular matrix protein that can inhibit EGFR signalling and affect fibrillogenesis | Binds the EGFR extracellular domain and inhibits dimerisation | TGF-β inhibition, ECM regulation | 0.999 |
| \| **HBEGF** \| \| --- \| | Heparin-binding EGF-like Growth Factor | EGFR ligand that mediates mitogenic Signalling via EGFR and ERBB2/4 | High-affinity ligand binding to EGFR/ERBB receptors | EGFR-mediated cell growth, wound healing | 0.999 |
| \| **EREG** \| \| --- \| | Epiregulin | EGFR/ERBB4 ligand that activates downstream phosphorylation cascades | Stimulates EGFR and ERBB4 dimerisation and Signalling | Proliferation, inflammation, and cancer progression | 0.999 |
| \| **CDH1** \| \| --- \| | Cadherin-1 (E-cadherin) | Calcium-dependent adhesion molecule; suppresses invasion and metastasis | Regulates EGFR internalization and localization | Cell-cell adhesion, tumour suppression | \| 0.999 \| \| --- \| |
| \| **GAB1** \| \| --- \| | GRB2-associated Binding Protein 1 | Scaffold protein that amplifies PI3K and MAPK Signalling downstream of EGFR | Binds to the GRB2-SHC complex post-EGFR activation | PI3K/AKT, MAPK Signalling | 0.999 |
| \| **PIK3CA** \| \| --- \| | Phosphoinositide-3-kinase Catalytic Subunit Alpha | Produces PIP3, promoting AKT activation | Binds activated EGFR via SH2 domain-containing adaptors | PI3K/AKT pathway, cell survival | 0.999 |
| \| **CBL** \| \| --- \| | E3 Ubiquitin-protein Ligase CBL | The tags activated EGFR for endocytosis and degradation | Ubiquitinates EGFR at tyrosine residues | Receptor downregulation, EGFR trafficking | \| 0.999 \| \| --- \| |
| \| **EGF** \| \| --- \| | Epidermal Growth Factor | The canonical ligand of EGFR initiates receptor activation | Binds to the extracellular domain of EGFR | Cell proliferation, migration, survival | 0.999 |
| \| **ERBB3** \| \| --- \| | Erb-B2 Receptor Tyrosine Kinase 3 | Forms inactive homodimers but active heterodimers with EGFR or ERBB2 | Heterodimerisation with EGFR enhances signal strength | PI3K/AKT, neuregulin Signalling | \| 0.999 \| \| --- \| |
| \| **ERBB2** \| \| --- \| | Erb-B2 Receptor Tyrosine Kinase 2 (HER2) | Coreceptor lacking ligand-binding domain, augments EGFR Signalling | Potent heterodimer partner for EGFR | Breast cancer, cell proliferation pathways | 0.999 |

**Table S5: Detailed Overview of Predicted ALK Functional Partners**

| **Protein** | **Full Name** | **Function** | **Biological Significance** | **Score** |
| --- | --- | --- | --- | --- |
| **EML4** | Echinoderm Microtubule-Associated Protein-Like 4 | Modifies the assembly dynamics of microtubules | Known fusion partner of ALK in NSCLC, driving oncogenic Signalling | **0.999** |
| **PTN** | Pleiotrophin | Secreted growth factor that signals via non-proteoglycan receptors | Activates ALK, promotes cell proliferation, angiogenesis, and tumour growth | **0.999** |
| **MDK** | Midkine | Cytokines and growth factors bind to cell-surface proteoglycans | Overexpressed in several cancers, involved in ALK-mediated mitogenic pathways | **0.997** |
| **NPM1** | Nucleophosmin | Involved in ribosome biogenesis, centrosome duplication, and protein chaperoning | Oncogenic translocation partner in leukaemia; regulates cell stress and proliferation | **0.994** |
| **KRAS** | GTPase KRas | Binds GDP/GTP; key signal transducer in the RAS/MAPK pathway | Frequently mutated in cancers, integrates ALK Signalling into downstream proliferation cascades | **0.989** |
| **NRAS** | GTPase NRas | Similar to KRAS, it possesses intrinsic GTPase activity | Mutated in melanoma and hematologic malignancies, connected to ALK-activated pathways | **0.986** |
| **PIK3CA** | Phosphatidylinositol 4,5-bisphosphate 3-kinase catalytic subunit alpha | PI3K enzyme phosphorylating PIP2 to PIP3 | Major component of the PI3K-AKT pathway; promotes survival, growth under ALK activation | **0.979** |
| **PLCG1** | 1-phosphatidylinositol 4,5-bisphosphate phosphodiesterase gamma-1 | Produces IP3 and DAG, mediating second messenger Signalling | Facilitates Ca²⁺-dependent Signalling in response to ALK and RTK activation | **0.977** |
| **PIK3R1** | PI3K regulatory subunit alpha | Binds phosphorylated RTKS, stabilising PIK3CA activity | Regulates PI3K-AKT pathway; contributes to drug resistance in ALK-positive tumours | **0.973** |
| **ALKAL2** | ALK and LTK Ligand 2 | Ligand for ALK and LTK | Stimulates ALK Signalling; essential in neuronal development and neuroblastoma biology | **0.968** |

**Table S6: Detailed Overview of Predicted KRAS Functional Partners**

| **Gene Symbol** | **Protein Name** | **Function/Description** | **Score** | **Evidence Sources** |
| --- | --- | --- | --- | --- |
| **RALGDS** | Ral guanine nucleotide dissociation stimulator | Promotes GDP-GTP exchange on RalA and RalB; links KRAS to Ral Signalling. | 0.999 | Experiments, Text Mining, Databases |
| **SOS1** | Son of sevenless homolog 1 | Facilitates GDP-GTP exchange on KRAS; promotes Ras activation. | 0.999 | Experiments, Databases, Text Mining |
| **RAF1** | RAF proto-oncogene serine/threonine-protein kinase | Mediates downstream MAPK Signalling from KRAS; controls proliferation and differentiation. | 0.999 | Experiments, Databases, Text Mining |
| **BRAF** | Serine/threonine-protein kinase B-Raf | Kinase involved in MAPK/ERK Signalling downstream of KRAS. | 0.999 | Experiments, Text Mining, Databases |
| **PIK3CA** | Phosphatidylinositol 4,5-bisphosphate 3-kinase catalytic subunit alpha | Central node of the PI3K pathway; transduces survival signals from KRAS. | 0.998 | Experiments, Databases, Text Mining |
| **CALM3** | Calmodulin 1 | Controls the activity of kinases, ion channels, and other enzymes via calcium binding. | 0.998 | Coexpression, Text Mining, Homology |
| **CALML6** | Calmodulin-like protein 6 | Member of the calmodulin family; involved in calcium Signalling and cellular regulation. | 0.998 | Text Mining, Coexpression |
| **CALML3** | Calmodulin-like protein 3 | May act as a light chain for unconventional myosin-10; roles in calcium-dependent processes. | 0.998 | Text Mining, Coexpression |
| **CALML5** | Calmodulin-like protein 5 | Binds calcium; may regulate keratinocyte differentiation. | 0.998 | Text Mining |
| **CALML4** | Calmodulin-like protein 4 | Calcium-binding protein; part of the calmodulin family. | 0.998 | Text Mining |

**Table S7: Detailed Overview of Predicted PD-1 Functional Partners**

| **Gene Symbol** | **Protein Name** | **Function/Description** | **Score** | **Evidence Sources** |
| --- | --- | --- | --- | --- |
| **CD274** | Programmed cell death ligand 1 (PD-L1) | Ligand for PD-1; suppresses immune response and promotes tolerance. | 0.999 | Databases, Experiments, Text Mining |
| **PDCD1LG2** | Programmed cell death ligand 2 (PD-L2) | Alternative ligand for PD-1; contributes to IFN-γ production and immune suppression. | 0.999 | Databases, Experiments, Text Mining |
| **CD80** | T-lymphocyte activation antigen CD80 | Costimulatory molecule; modulates T-cell proliferation and immune response. | 0.998 | Experiments, Databases |
| **PTPN11** | Tyrosine-protein phosphatase non-receptor type 11 (SHP2) | Dephosphorylates TCR Signalling proteins upon PD-1 engagement. | 0.998 | Experiments, Text Mining |
| **CD86** | T-lymphocyte activation antigen CD86 | Costimulatory receptor; essential for T-cell activation and proliferation. | 0.996 | Experiments, Databases, Text Mining |
| **CTLA4** | Cytotoxic T-lymphocyte-associated protein 4 | Negative regulator of T-cell immune responses; shares ligands CD80 and CD86 with CD28. | 0.992 | Experiments, Databases |
| **LGALS9** | Galectin-9 | Binds to HAVCR2/TIM3; modulates T cell death and immunoregulation. | 0.969 | Text Mining, Coexpression |
| **PTPN6** | Tyrosine-protein phosphatase non-receptor type 6 (SHP1) | Modulates TCR Signalling via PD-1-mediated recruitment. | 0.961 | Text Mining, Coexpression |
| **CD4** | T-cell surface glycoprotein CD4 | Coreceptor in TCR Signalling; modulates immune responses. | 0.960 | Text Mining, Coexpression |
| **LAG3** | Lymphocyte activation gene 3 protein | Inhibitory receptor contributes to immune checkpoint regulation. | 0.950 | Text Mining, Coexpression |
